# Building the Case for Insulin-Like Growth Factor Receptor-I Involvement in Thyroid-Associated Ophthalmopathy

**DOI:** 10.3389/fendo.2016.00167

**Published:** 2017-01-03

**Authors:** Terry J. Smith, Joseph A. M. J. L. Janssen

**Affiliations:** ^1^Department of Ophthalmology and Visual Sciences, Division of Metabolism, Endocrinology, and Diabetes, University of Michigan Medical School, Ann Arbor, MI, USA; ^2^Department of Internal Medicine, Division of Metabolism, Endocrinology, and Diabetes, University of Michigan Medical School, Ann Arbor, MI, USA; ^3^Department of Internal Medicine, Erasmus Medical Center, Division of Endocrinology, Rotterdam, Netherlands

**Keywords:** autoimmune, insulin-like growth factor I receptor, thyrotropin receptor, Graves’ disease, hybrid receptor, antibodies, autoantibodies

## Abstract

The pathogenesis of orbital Graves’ disease (GD), a process known as thyroid-associated ophthalmopathy (TAO), remains incompletely understood. The thyrotropin receptor (TSHR) represents the central autoantigen involved in GD and has been proposed as the thyroid antigen shared with the orbit that could explain the infiltration of immune cells into tissues surrounding the eye. Another cell surface protein, insulin-like growth factor-I receptor (IGF-IR), has recently been proposed as a second antigen that participates in TAO by virtue of its interactions with anti-IGF-IR antibodies generated in GD, its apparent physical and functional complex formation with TSHR, and its necessary involvement in TSHR post-receptor signaling. The proposal that IGF-IR is involved in TAO has provoked substantial debate. Furthermore, several studies from different laboratory groups, each using different experimental models, have yielded conflicting results. In this article, we attempt to summarize the biological characteristics of IGF-IR and TSHR. We also review the evidence supporting and refuting the postulate that IGF-IR is a self-antigen in GD and that it plays a potentially important role in TAO. The putative involvement of IGF-IR in disease pathogenesis carries substantial clinical implications. Specifically, blocking this receptor with monoclonal antibodies can dramatically attenuate the induction by TSH and pathogenic antibodies generated in GD of proinflammatory genes in cultured orbital fibroblasts and fibrocytes. These cell types appear critical to the development of TAO. These observations have led to the conduct of a now-completed multicenter therapeutic trial of a fully human monoclonal anti-IGF-IR blocking antibody in moderate to severe, active TAO.

## Introduction

The mechanisms underlying Graves’ disease (GD) remain incompletely understood (1). Among the open questions is the basis for loss of immunological tolerance to the thyrotropin receptor (TSHR). Factors underpinning the orbital manifestations of GD, a process known as thyroid-associated ophthalmopathy (TAO), are even less well understood. The unambiguous identification of a pathogenic autoantigen shared by the orbit and thyroid gland remains to be accomplished. TSHR is the most likely candidate by virtue of its established central role in mediating the hyperthyroidism associated with GD. It has been detected, albeit at very low levels, in the healthy orbit and at somewhat higher levels in orbital tissues during TAO (2). Thyroglobulin (Tg) is another antigen suspect because of its previously unexplained presence in the diseased orbit (3). The insulin-like growth factor-I receptor (IGF-IR) has joined the conversation. It appears to be overexpressed in GD in several cellular compartments (4). Insinuation of IGF-IR in TAO has ignited substantial debate among workers in the field of thyroid autoimmunity (5, 6). In this brief review, we attempt to present a balanced assessment of evidence both refuting and supporting the concept that IGF-IR plays an active and important disease-promoting role in TAO. We also review the proposed mechanisms through which the receptor might serve as a molecular conduit for transducing disease-related signaling initiated by IGF-IR itself and by TSHR. It is possible that IGF-IR might be effectively targeted as therapy for TAO.

## General Concepts about the IGF-IR

IGF-IR and the insulin receptor (IR) belong to the family of ligand-activated, plasma membrane-bound tyrosine kinase receptors. Both receptors are widely expressed in many tissues (7). They exhibit substantial structural homology. Depending on which regions are compared, they share sequence identities varying from 41 to 84% (8). Nevertheless, they serve distinct physiological functions *in vivo* (9). Because IGF-I and insulin can produce the same biological responses, and given the wide-spread tissue distribution of IGF-IR and IR, it has been difficult to determine which of these two receptors mediates a particular response (10). Separation of the different physiological functions mediated through these receptors *in vivo* is imposed by several factors, including their tissue distribution (9). While IR is primarily involved in metabolic actions, IGF-IR promotes cell survival, growth, and differentiation (9). However, IGF-I and insulin can interact promiscuously through both receptors, although with substantially different affinities (11).

IGF-IR like IR comprises two extracellular α-subunits, each containing an IGF-I binding site, and two trans-membrane β-subunits where the catalytic determinants for intrinsic tyrosine kinase activity are located (7). IGF-I elicits multiple biological responses through its high-affinity binding to IGF-IR. Transduction of IGF-I-provoked signaling is initiated through activation of the intrinsic tyrosine kinase and autophosphorylation of IGF-IR. This results in the phosphorylation of multiple tyrosine-containing downstream substrates, including the IRS and Shc proteins (12). Differences in interactions with these substrates arise from the divergent structures of β-subunit and kinase domains in IGF-IR and IR. These variations are hypothesized as being partially responsible for IGF-I and insulin specificity (13). Activated ligand-receptor complexes are thought to be internalized into endosomes (14). Specificity of IGF-I and insulin *in vivo* may result from divergence in the levels of the respective receptors in target tissues coordinated with respective ligand concentration and availability (15). Hybrid receptors comprising both IGF-IR and IR may form in cells expressing both proteins (16). These hybrids are formed during the normal posttranslational processing of both receptors (16). Their formation appears to be stochastic and is therefore receptor concentration-dependent (17). They also appear to determine relative responsiveness to IGF-I and insulin. When levels of IGF-IR exceed those of IRs, IR monomers are mainly present as hybrid receptors (17). These hybrids exhibit high affinity for IGF-I and thus shift the bias away from insulin responsiveness. Although the functional role of hybrid receptors remains incompletely understood, studies have demonstrated that they behave more like IGF-IRs than IRs (16). IGF-IR can also heterodimerize with receptors belonging to other families (18). For example, it can form heterodimers with epidermal growth factor (19). Inhibition of one constituent of these hybrids can shift signaling toward its counterpart receptor (18).

By virtue of its catalytic domain, IGF-IR has traditionally been considered a member of the receptor tyrosine kinase family. It appears that receptor autophosphorylation, particularly at tyrosine residues 1131, 1135, and 1136, is critical to initiation of IGF-I-dependent signaling (20, 21). However, this concept of IGF-IR activation appears to be oversimplified (22). A revised model has now been developed to explain how IGF-I or other activating ligands initiate IGF-IR internalization and subsequent degradation through lysosomal or proteasomal pathways (23). Evidence supports β-arrestins, already implicated in the regulation of G protein-coupled receptors (GPCRs), serving as adaptors between the oncoprotein, E3 ubiquitin ligase Mdm2, and IGF-IR (24). Mdm2 was originally described as controlling IGF-IR ubiquitination and in so doing, promoting its degradation by the proteasome system (25). In this manner, β-arrestin-1 acts as a crucial component in IGF-IR ubiquitination and downregulation. On the other hand, recent studies provide evidence that IGF-IR ubiquitination by β-arrestins/Mdm2 is not simply a receptor desensitization system. While down-regulating IGF-IR from the cell surface and inhibiting its “classical” kinase signaling, β-arrestins activate alternative signaling through MAPK (26). The roles played by β-arrestin-1 in IGF-IR resemble its functions in regulating the behavior of GPCRs. Thus the protein suppresses IGF-IR activity while promoting MAPK signaling (22, 27).

## General Concepts about the TSHR

It has been more than 40 years since convincing evidence was put forward for a cell surface-displayed TSHR on thyroid epithelial cells (28). The TSHR gene was first cloned by Vassart and colleagues in 1989 (29). The encoding mRNA has been detected subsequently not only in thyroid tissue but also in multiple fatty depots in animals and human beings (30, 31). Its cognate ligand, TSH’ is a glycoprotein hormone produced by thyrotrophs located in the anterior pituitary gland. TSHR plays a central role in the regulation of thyroid growth and function (32). More recently, the receptor was co-crystalized with anti-TSHR antibodies and its structure solved (33, 34). TSHR belongs to the family of rhodopsin-like GPCRs which also includes receptors for luteinizing hormone (LH) and follicle-stimulating hormone (FSH). These proteins possess seven plasma membrane-spanning regions within the so called serpentine domain (35). Surface-displayed TSHR exists as a multimeric structure (36). The extracellular region represents the amino-terminus containing a high-affinity TSH binding site. The unligated extracellular domain interacts as an inverse agonist with the serpentine domain. TSHR is encoded by a single gene and is synthesized as a single peptide chain that undergoes cleavage into “A” and “B” subunits (37). These are then linked by a disulfide bond. Unlike the receptors for LH and FSH, the extracellular TSHR domain undergoes metalloproteinase-dependent cleavage (38). Some debate exists as to whether the cleavage occurs at the same precise site(s) on the protein and whether the resulting C-peptide sequence is invariant. The specific protease responsible for this cleavage has yet to be identified (39). Evidence has been introduced supporting the concept that this cleaved receptor fragment is shed and provokes the generation of thyroid-stimulating IgGs (TSI) (40). Some authors have expressed the view that the cleaved fragments of TSHR are released into thyroid lymphatics draining into lymph nodes where they are processed by antigen-presenting cells through interactions with mannose receptors (38). TSIs are responsible for the hyperthyroidism associated with GD (40). But not all anti-TSHR antibodies are stimulatory. Some block binding of TSH to the receptor (33) while others are viewed as “neutral.” The exact mechanisms involved in the activation of TSHR by either TSH or TSIs remain uncertain although the ligand binding epitopes have been localized (33, 34). Interactions between the different classes of anti-TSHR antibodies and the receptor have also been characterized (41). Signaling downstream from TSHR is complex and involves several pathways that cross talk in patterns that determine the ultimate genes targeted for activation (42–44). Similar but non-identical downstream signaling occurs following TSH and TSI binding to TSHR (45).

## Extra-Thyroidal TSHR

Detection of TSHR expression peripheral to the thyroid gland has implicated the protein in an expanding array of biological functions. Particular focus on extra-thyroidal TSHR has involved studies examining the pathogenesis of TAO. Feliciello et al. detected TSHR mRNA in orbital tissues from healthy donors and those with GD (2). TSH promotes lipolysis in rodents and human beings (46, 47). With more advanced techniques of detection, TSHR has been identified, albeit at a very low level, in many fatty and non-adipose tissues (48). The receptor has recently been insinuated in the regulation of bone metabolism (49).

## Evidence for Interactions Between IGF-IR and TSHR

Accumulating evidence supports the general concept that dissimilar receptor proteins can interact by forming complex signaling partnerships. Recently, Girnita et al. suggested that IGF-IR forms functional hybrids with GPCRs (27). These hybrids utilize components of GPCR signaling and can thus activate pathways conventionally used by GPCRs (27). Multimeric molecular structures of these receptor complexes may help explain the functional interplay that appears to occur between IGF-IR and TSHR pathways. A relationship between IGF-I and TSH signaling was first recognized in 1986 by Ingbar and colleagues (50). They demonstrated that IGF-I could either enhance or antagonize the actions of TSH in cultured thyroid epithelial cells. For instance, IGF-I facilitates the actions of TSH on FRTL-5 cell proliferation while attenuating its induction of sodium/iodide symporter, interactions mediated through PI3 kinase (51). A synergy between the two factors was further demonstrated in the induction of 1, 2-diacylglycerol production in rat thyroid epithelium. In thyroid, the mitogenic activity of IGF-I can be potentiated by TSH (52, 53). TSH induces IRS-2 monoubiquitination in cultured thyroid cells, thereby enhancing IGF-I signaling and mitogenic activity. Both TSH and IGF-I enhance the nuclear content of β-catenin and thus promote Wnt-dependent thyroid epithelial proliferation (54). Conditional knock-out of IGF-IR in thyroid tissue results in increased serum TSH levels and lower serum thyroxine concentrations (55). This profile of circulating hormones suggests relative TSHR insensitivity. In contrast, over-expression of IGF-IR in thyroid amplifies the action of TSH and exaggerates its impact on the synthesis of thyroid hormones (56). We hypothesize that a similar potentiating mechanism might apply following TSHR stimulation by circulating TSI. Further studies will be required to determine whether such a mechanism might underlie the results found in some actions of TSI in the pathogenesis of TAO.

It was uncertain how the two pathways might cross talk at the target cellular level until Tsui and colleagues reported that TSHR and IGF-IR appear to interact directly by forming a protein complex (57). Evidence for these TSHR/IGF-IR complexes was found in orbital fibroblasts and thyroid epithelium utilizing several strategies including co-localization studies with confocal microscopy and co-immunoprecipitation assays. Tsui et al. further demonstrated that a monoclonal blocking antibody directed against IGF-IR could attenuate activation of Erk1/2 by IGF-I, rhTSH, and IgG from patients with GD (57). This report unambiguously demonstrated the functional interdependence of TSHR and IGF-IR and strongly suggested that IGF-IR was transactivated by TSHR. It was followed by several papers confirming (58) and in some cases extending (59, 60) these observations. Evidence for bidirectional crosstalk between the two receptors was demonstrated in another study in orbital fibroblasts (60). IGF-I and TSH were shown to act synergistically in that study by inducing HA production in orbital fibroblasts. Another recent paper contained evidence that inhibiting PI3 kinase and mTOR could attenuate HA accumulation upregulation mediated by these receptors (61). Unfortunately, cultures were exposed to the small molecule inhibitors for many days, inviting criticism of the study design used where conclusions were drawn based on findings that may have been non-specific. Another recent report demonstrated dependence on TSHR in TSHR knock-out mice of IGF-IR protein distribution and levels (62).

## Evidence for Involvement of IGF-IR in TAO

Whether a specific autoantigen(s) shared by the orbit and thyroid participates in the pathogenesis of TAO remains an open question. To our knowledge, demonstration of antigen-specific T cells among those lymphocytes infiltrating the orbit has yet to be unambiguously accomplished. One of the earliest investigators to explore the issue of an ectopically expressed thyroid antigen in the orbit was Kriss (3). He and his colleagues detected Tg in the TAO orbit using thyroidolymphography over four decades ago. More recent studies have substantiated this earlier work (63). Anti-Tg antibodies are commonly detected in thyroid autoimmunity including a substantial proportion of those individuals with GD; however, it is unclear how Tg or the antibodies directed against this protein might play an active role in TAO.

The IGF-I pathway was first implicated in TAO by Weightman et al. (64) who detected immunoglobulins in the sera of individuals with TAO that could displace binding of radiolabeled IGF-I from orbital fibroblast monolayers. This important study was the first to question whether antibodies directed against an IGF-I binding site might be present in these patients. Later studies from Pritchard et al. (65, 66) reported similar results and identified the binding site on orbital fibroblasts as IGF-IR. Their studies indicated that GD-IgG and IGF-I recognize a common binding site. These later studies also revealed that circulating IgGs in GD could induce chemokine expression in TAO orbital fibroblasts, indicating that at least some of these antibodies were biologically active. Pritchard et al. mapped the critical signaling downstream from IGF-IR to the FRAP/mTor/p70^S6k^ pathway. They further demonstrated that the induction of IL-16 and RANTES was inhibited by rapamycin and by transfecting cells with a dominant negative IGF-IR (65, 66). The report also provided evidence for IGF-IR over-expression in these cells when compared to the levels of the receptor in orbital fibroblasts from healthy tissue.

## Are Stimulatory anti-IGF-IR Antibodies Distinct from TSI?

Reports from Pritchard et al. (65, 66) and Smith and Hoa (67) suggested that IgGs circulating in patients with GD can activate orbital fibroblasts have proven to be controversial (5, 6). The debate rests on whether activating antibodies differing from those against TSHR (i.e., TSI) and instead directly targeting IGF-IR are responsible for the upregulation of cytokine expression and hyaluronan production in orbital fibroblasts (65–67). A major barrier to our quest for the definitive answer derives from an inability to distinguish antibodies that activate IGF-IR from those that merely bind the receptor but fail to initiate signaling. Among the strongest evidence that anti-IGF-IR antibodies are generated in GD are the observations of Weightman et al. (64) and Pritchard et al. (65) demonstrating that GD-IgGs displace IGF-I binding to orbital fibroblasts. More recently, TSHR A-subunit plasmid DNA immunization of mice was shown to result in generation of anti-IGF-IR antibodies (68). Those studies were unable to detect any additional effects of co-immunization with TSHR and IGF-1Rα plasmids on the animal phenotype (68). Thus none of these reports provides insight into whether the anti-IGF-IR antibodies, distinct from TSI, can activate the receptor. Some workers in the field attribute activities of GD-Ig to TSIs rather than IgGs targeting IGF-IR; however, subsequent studies by Pritchard et al. may provide some guidance (69). They demonstrated similar cytokine-inducing activity in synovial fibroblasts from patients with rheumatoid arthritis (RA) when challenged by RA-IgG (69). Their findings indicate that disease-specific IgGs apart from TSI are likely driving these inductions.

More recent studies examining whether activating anti-IGF-IR antibodies are generated in GD have yielded disparate results. Experiments conducted in undifferentiated orbital fibroblasts treated with rhTSH or GD-IgG failed to generate increased levels of HA (70). In contrast, once differentiated into adipocytes, these fibroblasts responded to both (71). Varewijck and colleagues (72) have detected activating anti-IGF-IR antibodies in subsets of patients with GD. They monitored the phosphorylation of multiple tyrosine residues of IGF-IR as the primary read-out for assessing IGF-IR activity (72). In contrast, Minich et al. (73) were unable to distinguish between low levels of anti-IGF-IR IgG activity in healthy controls and those with GD. Their assay was limited to detecting phosphorylation of a single adjacent pair of tyrosine residues (Tyr 1165/1166). They quantified the titer of IGF-IR autoantibodies but their assay was incapable of discriminating between activating and non-activating antibodies. Furthermore, their estimates of the lower limits of antibody titers were based on arithmetic arguments rather than on empirical observations. Another potentially confounding limitation of their study was the likely insensitivity of their assay to low-affinity antibodies. Moreover, effects of stimulating antibodies frequently occur within a narrow concentration range (74) and their studies did not investigate the impact of higher and lower antibody titers. In sum, the conclusions drawn by Minich et al. appear to ignore the likely complex relationship between circulating antibody titers and the magnitude of their biological effects.

Krieger et al. reported an induction by GD-IgG of hyaluronan release from orbital fibroblasts despite an absence of IGF-IR autophosphorylation (59). The authors argued that this scenario rules against an activation of IGF-IR occurring during this action of GD-IgG. They concluded that the actions of GD-IgG must, therefore, be initiated by TSHR rather than through direct interactions with IGF-IR. Yet the authors provided apparently contradictory evidence for receptor activation by demonstrating that the specific IGF-IR tyrosine kinase inhibitor, linsitinib, blocks induction by GD-IgG of hyaluronan production. Thus, we interpret their findings as strongly suggesting that the Western blot assay they used for monitoring IGF-IR phosphorylation failed to detect what might have been low-level but physiologically important receptor activation.

Factors potentially underlying these divergent results include the wide array of assays used, differing target cell types, and the culture conditions used. With regard to culture media, lot to lot variability of endogenous IGF-I, IGF-II, and IGFBP concentrations in the animal sera could alter the background read-out activities observed as well as the magnitude of cellular responses. Thus, it remains possible although unproven that two discrete antibodies generated in GD are at play in the pathogenesis of TAO. This theoretical construct involves one antibody directed at TSHR and the other at IGF-IR. Antibody-induced receptor activation might exhibit tissue specificity. Due to their relatively long half-life of greater than 1 week (52), antibody-dependent activation of TSHR and IGF-IR could be relatively long-lived. It should be stressed that all currently available *in vitro* systems for assessing effects of antibodies on cultured cells may fail to mimic conditions existing *in vivo*. This could result in inaccurate estimates of the events occurring *in situ* within the orbit and potentially in thyroid tissue. In any event, assessment of anti-IGF-IR antibodies activating pathways conventionally used by GPCRs is unprecedented until now.

**Figure 1:**
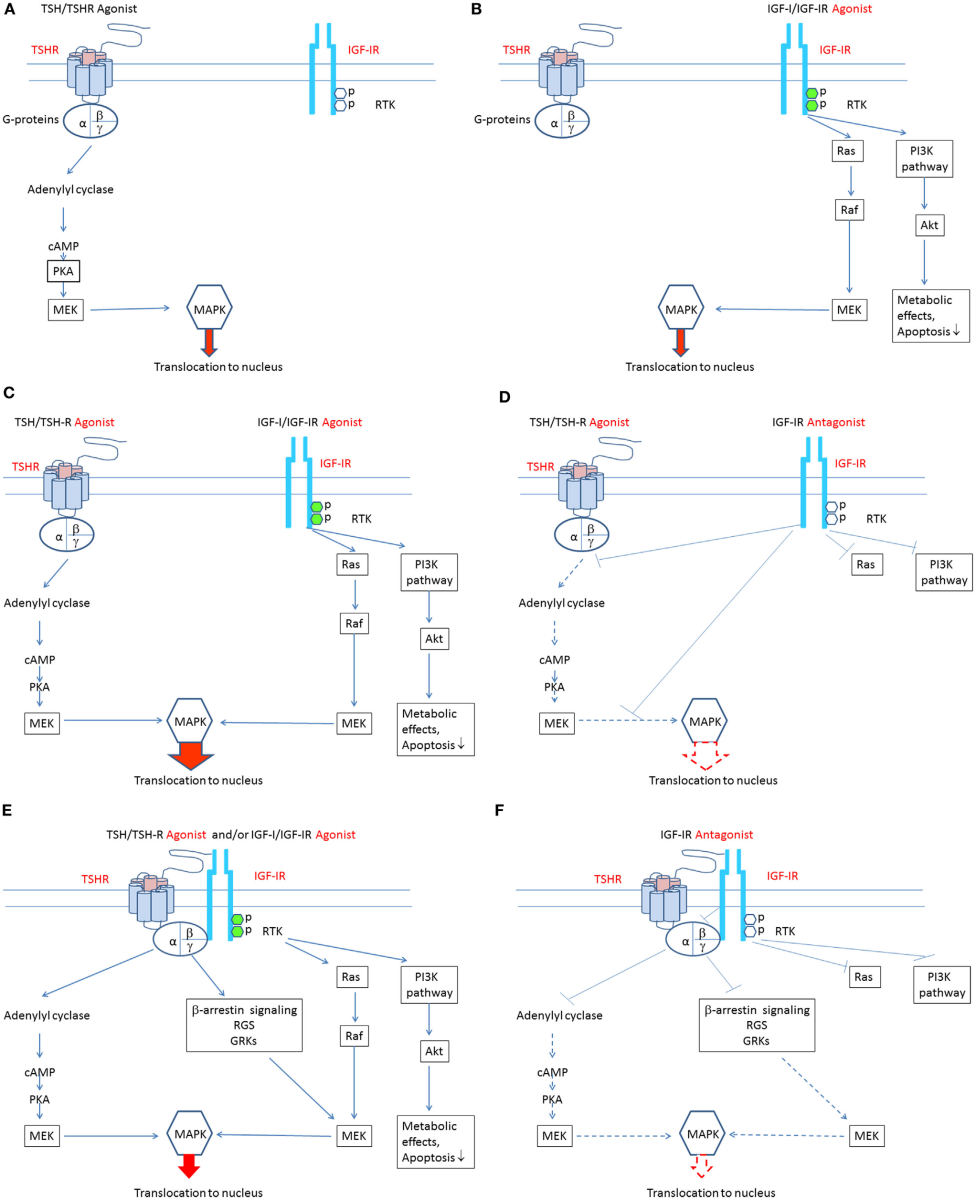
**Theoretical mechanisms involved in the crosstalk between insulin-like growth factor-I receptor (IGF-IR) and thyrotropin receptor (TSHR) pathways**. **(A)** Binding of TSH-like agonists to TSHR activates the classical post-receptor pathway by inducing cAMP production, resulting in activation of protein kinase A, mitogen-activated ERK kinase (MEK), and mitogen-activated protein kinase (MAPK). Phosphorylated MAPK translocates to the nucleus where it stimulates several transcription factors regulating gene expression. In this scenario, TSHR activation of its post-receptor pathways is independent of IGF-IR activation. **(B)** Binding of IGF-I-like agonists to IGF-IR activates the classical post-receptor pathway by inducing receptor autophosphorylation leading to activation of the phosphoinositide 3-kinase pathway and phosphorylation of Akt. Phosphorylated Akt increases translocation of glucose and is essential for cell survival. Auto-phosphorylated IGF-IR may also activate Ras which stimulates RAF kinase activity and that of MEK, leading to stimulation of mitogen-activated protein kinase (MAPK). Phosphorylated MAPK translocates to the nucleus where it phosphorylates specific transcription factors regulating gene expression. In this scenario, stimulation of IGF-IR and its post-receptor pathways is independent of TSHR activation. **(C)** Bidirectional crosstalk between the two receptors can occur. IGF-IR agonists can enhance the effects of TSHR agonists. When IGF-IR agonists bind to IGF-IR and TSHR agonists bind to TSHR, additive/synergistic effects can result in higher amplitude stimulation and phosphorylation of MAPK than that resulting from TSHR agonists or IGF-IR agonists acting alone. **(D)** A specific antibody directly targeting IGF-IR might attenuate both IGF-IR- and TSHR-mediated events, thus inhibiting additive/synergistic actions of IGF-IR agonists mediated through TSHR. Blocking IGF-IR with an IGF-IR-specific antagonist may be equivalent to its knockdown. This situation is accompanied by relative TSHR insensitivity (55). **(E)** IGF-IR and TSHR appear to form a physical/functional tyrosine kinase/G protein-coupled receptor (RTK/GPCR) hybrid (57). Such hybrids utilize components of GPCR signaling and can thus activate conventional pathways used by both receptors. Importantly, IGF-IR stimulation by IGF-IR agonists may lead to non-canonical TSHR signaling. Thus, the identical pathways downstream from TSHR may be activated. In this model, signaling downstream from TSHR may occur independently of TSHR activation. Thus, functional IGF-IR/TSHR hybrids may result in bidirectional receptor crosstalk. **(F)** Formation of IGF-IR/TSHR hybrid receptors may underlie inhibitory anti-IGF-IR antibody attenuation of actions initiated at both receptors. Thus, blocking IGF-IR may inhibit both IGF-IR and TSHR-mediated effects. This situation may carry functional equivalence to knocking down IGF-IR, where relative insensitivity to TSH has been demonstrated (55).

Most anti-IGF-IR antibodies target the ligand binding site and thus block the binding of endogenous ligands, thereby attenuating receptor activation (52). In contrast, antibodies binding elsewhere on the receptor may be more clinically relevant since they can induce receptor activation (74). Supporting this general concept is the observation that IR-stimulating antibodies activate the receptor by cross-linking subunits rather than by reacting to specific epitopes (74). Figures 1A–F summarize putative mechanisms involved in the pathogenesis of TAO. Agonists acting directly at both TSHR and IGF-IR may play roles in stimulating signaling pathways downstream from these receptors. Additional studies will be necessary to untangle what appear to be complex interactions that culminate in the disease.

## Ultimate Testing of the Hypothesis That IGF-IR Participates in TAO

Addressing the question of whether IGF-IR plays an important pathogenic role in TAO and thereby carries potential for therapeutic targeting must await studies conducted *in vivo*. That concept has been tested very recently in a multicenter, placebo controlled, double masked clinical trial of an IGF-IR blocking monoclonal antibody (teprotumumab or RV001) in active, moderate to severe TAO (http://clinicaltrials.gov/show/NCT01868997). The results of that prospective trial should shed new light on this as yet unresolved question.

## Author Contributions

TS reviewed the literature, drafted portions of the initial manuscript draft, amalgamated the portions of the paper generated by both authors, refined the text, and proof read the final draft. JJ reviewed the literature, drafted portions of the initial manuscript draft, refined the text, generated the theoretical model images, and proof read the final draft.

## Conflict of Interest Statement

TS holds patents related to the detection of antibody-mediated inflammatory autoimmune disorders (US 6936426), the diagnosis and therapy of antibody-mediated inflammatory autoimmune disorders (US 7998681 and US 8153121), and diagnostic methods related to Graves’ disease and other autoimmune disorders (US 8178304). No other potential conflict of interest relevant to this article was reported.
